# Frequent or scarce? Damage to flight–enabling body parts in bats (Chiroptera)

**DOI:** 10.1371/journal.pone.0219783

**Published:** 2019-07-22

**Authors:** Jan Cichocki, Marcin Warchałowski, Agnieszka Ważna, Iwona Gottfried, Anna Bator–Kocoł, Tomasz Gottfried, Adrianna Kościelska, Jacek Bojarski, Monika Pietraszko–Warchałowska, Grzegorz Gabryś

**Affiliations:** 1 Department of Zoology, University of Zielona Góra, Zielona Góra, Poland; 2 Department of Behavioural Ecology, University of Wrocław, Wrocław, Poland; 3 Polish Society of Wildlife Friends “pro Natura”, Wrocław, Poland; 4 Center for Applied Mathematics and Computer Science, Faculty of Mathematics, Computer Science and Econometrics, University of Zielona Góra, Zielona Góra, Poland; 5 Department of Invertebrate Biology, Evolution and Conservation, University of Wrocław, Wrocław, Poland; Museu de Ciències Naturals de Granollers, SPAIN

## Abstract

Bat wings are characterized by high endurance, and these mammals have developed a number of adaptations that protect them from falling into obstacles and potential injuries. However, in bat populations, there are individuals with visible fresh or healed injuries to the flight–enabling body parts. The aim of this research was to determine the differences in the occurrence of wing membrane damages among species of bats that differ in ecology and behavior. The study was conducted in southern and western Poland in the years 2000–2016 and included 3,525 individuals of six species: lesser horseshoe bat *Rhinolopus hipposideros*, Daubenton’s bat *Myotis daubentonii*, Natterer’s bat *Myotis nattereri*, greater mouse–eared bat *Myotis myotis*, western barbastelle *Barbastella barbastellus*, and brown long–eared bat *Plecotus auritus*. In all, 2.9% of the bats studied showed damage to the flight–enabling body parts. Natterer’s bat was the species with the highest number of injured individuals (21.74%). The lowest number of injured individuals (0.3%) was found in the brown long–eared bat. The most frequently observed type of damage was loss of an edge of the wing membrane (29.3%). The bat species studied differed significantly in the occurrence and location of flight enabling body parts damages. Certain behavioral and ecological factors like foraging mode, foraging habitats and habitat types of bat species determine the number of wing and tail membrane damages.

## Introduction

Bats and birds are the sole extant vertebrates with the ability to fly actively. Bat wings are different from bird wings in terms of their anatomical and morphological features [1]. Bat wings consist of a wing membrane (patagium), which is formed of a highly durable and flexible thin skin [2, 3]. The wing membrane is divided into three parts: propatagium, dactylopatagium, and plagiopatagium. Between the hind legs, there is a tail membrane called uropatagium. The surface area and the strength of the particular parts of the wing differ [3].

The size and the shape of the wings vary, even between closely related species. These qualities determine the maneuverability and the flight speed and thus, the hunting method and the food preferences of the bats [4, 5]. Narrow and long wings are characteristic of species flying fast and over long distances. Short and rounded wings are characteristic of species whose flight is slower but more agile [4]. In addition, the shape and the size of the uropatagium play a significant role during hunting by affecting flight agility and the way prey is captured [6, 7].

The causes and the types of damage to the flight–enabling body parts in bats have rarely been the subject of comprehensive research [8, 9]. Isolated cases of injuries were recorded, without detailed statistical summaries [7, 10–14]. Wing injuries, including the wing membrane, may be a result of predator attacks, aggressive behavior of other bats, or mechanical damage caused, for example, by bumping into vegetation, which may explain the thorns found in the wings [8]. The probability of injury occurrence is related to a number of factors, including the feeding method, foraging behavior, type of occupied hideout, and phenological period.

Losses in the wing membrane may also be a result of punching, a popular method of material acquisition, for example, for genetic tests. However, the results of certain studies on punching indicate that the losses of the wing membrane that occur this way heal quickly and do not leave permanent damage [15–17]. The fact that bats with holes in their wings are found in their natural habitats is used as the evidence of harmlessness of punching on the condition of bats [8, 18]. The injuries to the wings may also be a result of fungal disease like the white nose syndrome (WNS) that causes a greater or lesser necrosis in the wing membrane [19, 20].

Species of bats differ in terms of, for example, the method of echolocation and the hunting strategy. In our research, we assumed that ecological differences affect the frequency of the incidence of injuries to the wing membranes. We tested the following hypotheses: 1) species differ in terms of the proportion of individuals with damaged wings and tail membrane, 2) ecological differences, like foraging mode, foraging habitat and habitat type, determine the number of damages in wings and tail membrane, 3) foraging mode, habitat type and foraging habitat determine the differences in type and location of damage.

## Methods

We conducted this research in the lowland and mountainous areas of western and southern Poland from spring to autumn (excluding the winter hibernation period) in the years 2000–2016, in three different localities. The first was the Nietoperek nature reserve (52°23′22.7″N 15°29′07.4″E), a fortified region from the Second World War period, which is the biggest bat hibernaculum in Central Europe [21]. Observations were conducted in the years 2015–2016, during morphometric studies on three bat species (Daubenton’s bat *Myotis daubentonii*, Naterer’s bat *Myotis nattereri*, and greater mouse–eared bat *Myotis myotis*). The second was the wintering site in Chłodnia (ice house) in Cieszków (51°37′25.1″N 17°21′52.6″E) where observations were conducted in the years 2000–2016, during the ringing of three bat species (brown long–eared bat *Plecotus auritus*, western barbastelle *Barbastella barbastellus*, and greater mouse–eared bat) in the swarming period. The third study area was Silesian Beskids in the years 2014–2016, near three caves: Wiślańska Cave (18°57′26.40″N 49°39′54.70″E), Grabowa Cave (18°57′12.41″N 49°40′28.59″E), Cave in Stołów (18°58′33.00″N 49°44′28.00″E), and in the nursery colony in Grodziec (49°48′07.7″N 18°51′58.7″E), where observations were conducted during ringing and radio telemetry on the lesser horseshoe bat *Rhinolophus hipposideros*. The selection of species whose injuries are listed in this work was dictated by the type of research conducted. Bats were caught in mist nets at the entrances or inside their roosts. In all the cases, bats were carefully handled and thoroughly examined. The individuals were marked with a non–durable medical marker on plagiopatagium of right wing, and some were ringed. For individuals with injuries, the injuries were photo–documented. After taking the photographs, we released the individuals.

**Fig 1:**
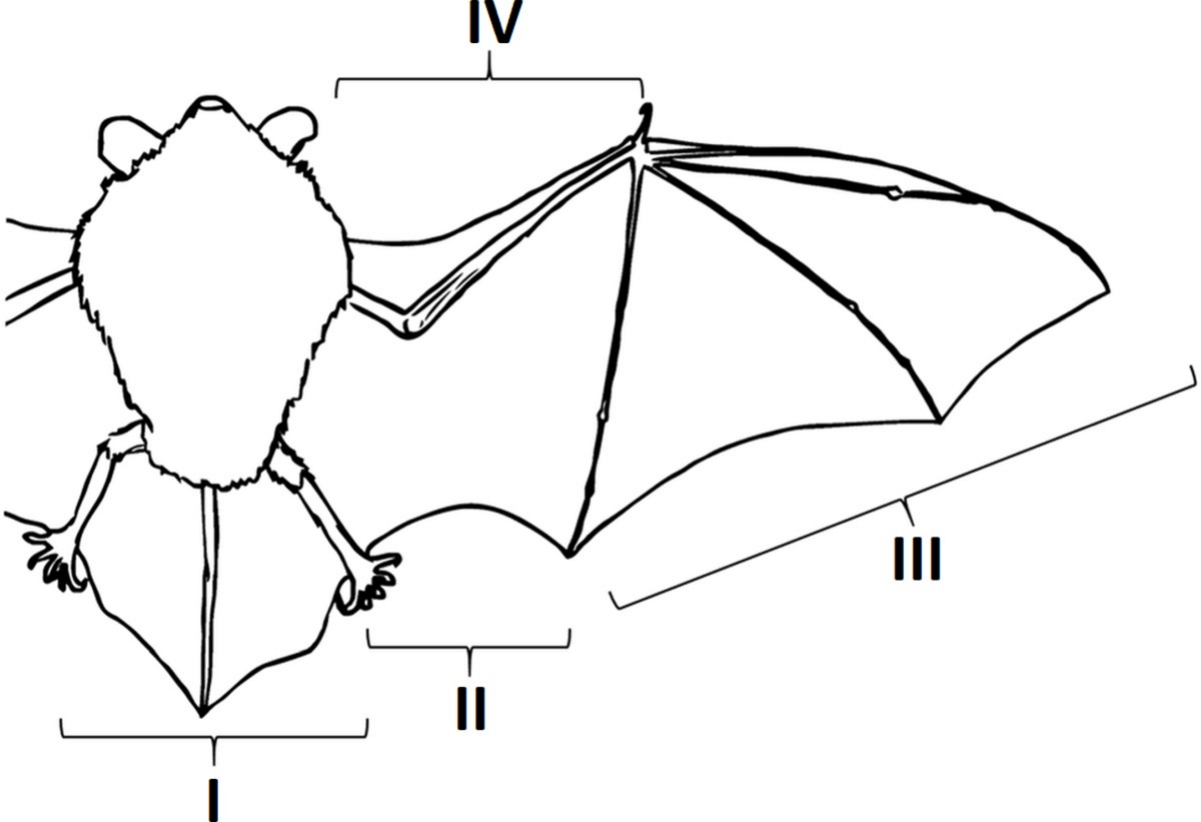
Division of the wing and the tail membrane applied in the present study. I–tail membrane, uropatagium, II–wing membrane, plagiopatagium, III–finger membrane, dactylopatagium, IV–propatagium.

**Fig 2:**
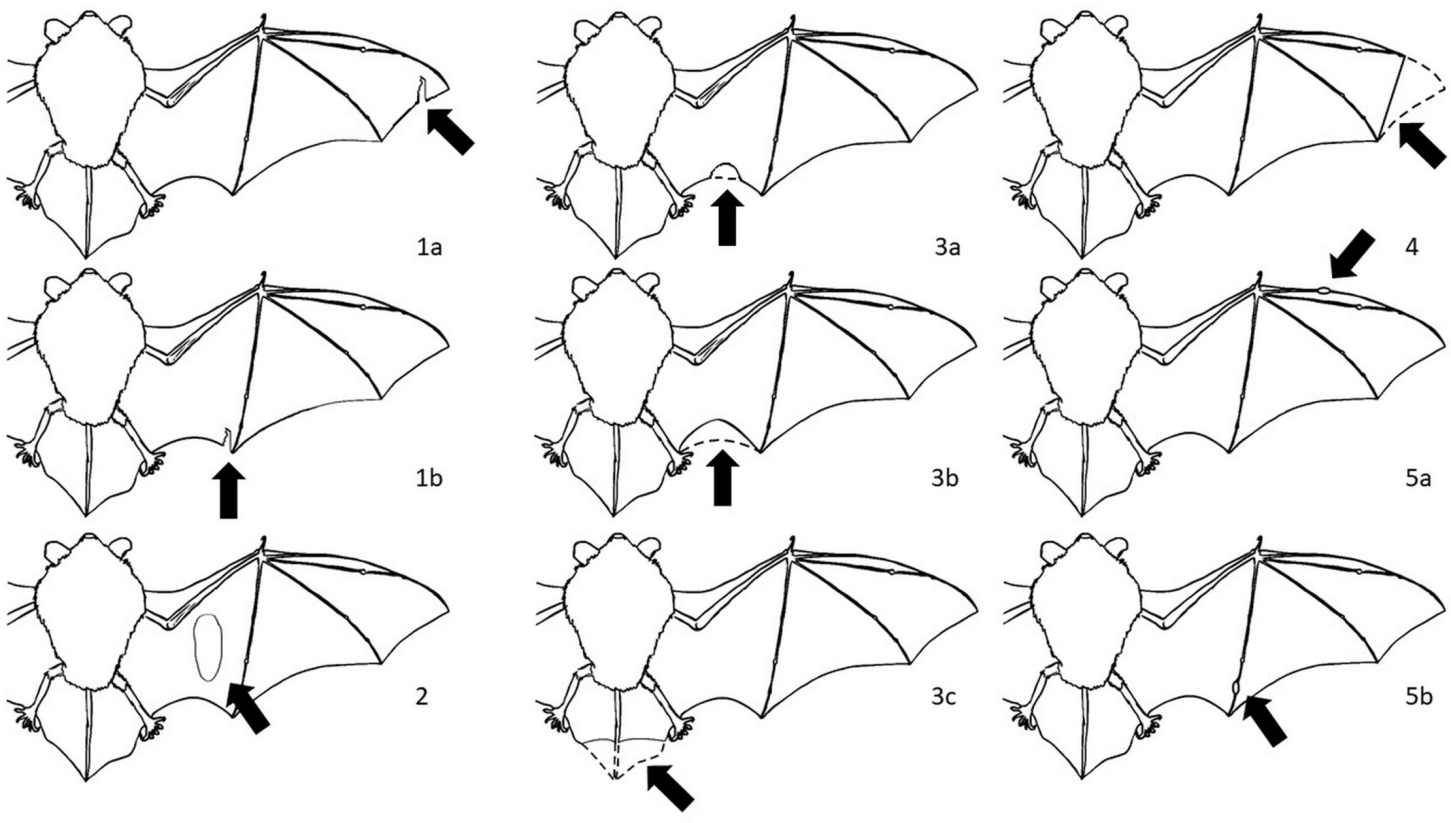
Damages to the wing membranes. 1 (a, b)–tears in the wing–disruption of the wing membrane running from its edge; 2 –holes in wings–holes with a diameter larger than 1 mm, damage to the wing membrane; 3 (a, b, c)–losses in the wing and tail membrane–losses of the edge fragments of the wing membrane, including scarrs that influence the shape of the wing and the lost fragments of uropatagium; 4 –loss of a finger membrane–damage to the dactylopatagium, and loss or deformation of the end part of the wing. 5 (a, b)–bone fractures–visible traces of healed injuries or bone fractures, such as bone thickening, bone fusion, and fractures with the displacement of metacarpal and phalanges bones.

On the basis of the observations and the analysis of the photographic material of the damages to the wing membranes, we categorized the damages according to their locations, i.e., the particular part of membrane based on the anatomical division: I–tail membrane (uropatagium), II–wing membrane (plagiopatagium), III–finger membrane (dactylopatagium), IV–propatagium (Fig 1). The injuries were divided into five groups according to the type of damage (Fig 2).

### Habitat and foraging mode

**Fig 3:**
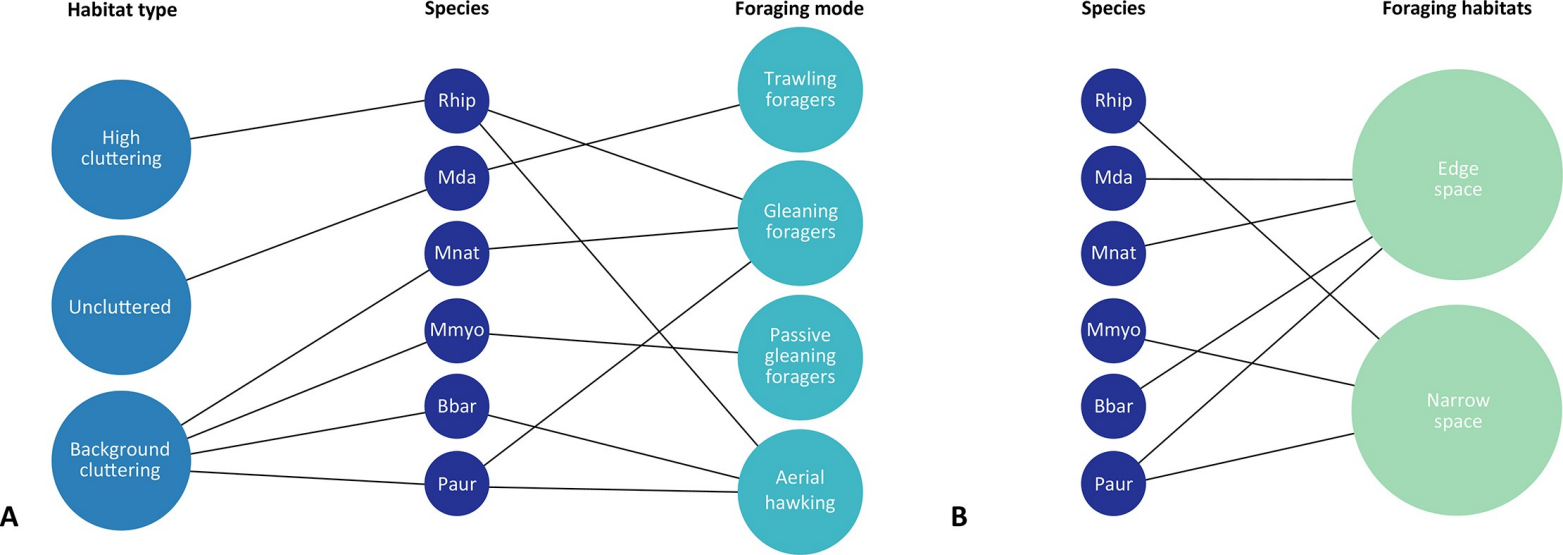
**Type of habitats occupied by bats, the corresponding foraging mode (A), and foraging habitat (B).** The abbreviation name: Rhip–*Rhinolophus hipposideros*, Mdau–*Myotis daubentonii*, Mnat–*Myotis nattereri*, Mmyo–*Myotis myotis*, Bbar–*Barbastella barbastellus*, Paur–*Plecotus auritus*.

The application of unequivocal division criteria such as habitat type, foraging mode, and the foraging habitats of bats is challenging, as bats can periodically change both their habitat and their foraging mode [22]. The applied division included the type of habitat that was most often occupied by specific bat species, the corresponding foraging mode, and the corresponding foraging habitat [22, 23] (Fig 3).

### Statistical analysis

We calculated the proportion of individuals with wing injuries in relation to the total number of bats studied and in relation to the number of individuals of each species. We estimated the probability of wing injuries in the studied species. We calculated the percentage of damage to particular parts of the wing and studied for any differences between the considered species in this respect. We calculated the percentage of bats with every type of damage within every examined species.

To test first hypothesis we grouped bats with and without wing and tail membrane damages within every species. We estimated the probability of occurrence of damage in certain species using the analysis of general linear models (GLM) [24]. Before using GLM models every categorical variable was transformed into dichotomous variables, having regard to their value.

We analyzed damages of flight enabling body parts in relation to foraging mode, habitat type and foraging habitat [22, 23]. We distinguished four foraging modes: a) trawling foragers b) gleaning foragers c) passive gleaning foragers d) aerial hawking (Fig 3); three habitat types: a) high cluttering b) uncluttered c) background-cluttering; and two foraging habitat types: a) edge space b) narrow space.

To test second hypothesis we have conducted an analysis using general linear models (GLM) with a logit link function for estimation of the probability of damage occurrence. To test third hypothesis we used Pearson's χ^2^ test to establish relation between foraging mode, habitat type and type of damage.

For analysis of relations between damages and foraging mode we used only data describing wing membrane and finger membrane (II and III). We omitted other parts because of too little sample. We analyzed data using general linear models (GLM) with a logit link function for estimation of the probability of damage occurrence.

For all the statistical tests, we used the significance level *p* = 0.05. All statistical analyses were conducted using R program [25] with default packages.

### Permits

We conducted the research on species protected by the Polish law on the basis of the derogations from the prohibitions, including the capture and temporary detention for performing the biometric measurements of bats in the area of the Nietoperek nature reserve, permit number WPN–I–6205.50.2015.AI issued by Directorate for Environmental Protection (RDOŚ) in Gorzów Wielkopolski, in the area of the Silesian Beskids permits number WPN.6401.106.2015.DC, and WPN.6401.379.2015.DC issued by Directorate for Environmental Protection (RDOŚ) in Katowice, in the area of Lower Silesia, permits number DOPweg–4201–04A–4/03/al issued by Polish Ministry of the Environment and WPN.6401.164.2015.IW issued by Directorate for Environmental Protection (RDOŚ) in Wrocław.

## Results

The study included 3,525 individuals of six species (S1 Appendix): *B*. *barbastellus*– 2,376; *P*. *auritus*– 333; *R*. *hipposideros*– 130; *M*. *myotis*– 394; *M*. *daubentonii*– 200; *M*. *nattereri*– 92. In all, during the research, we found 105 individuals with various types of damage to wing membranes and wings. Bats with injuries accounted for 2.9% of all the individuals studied. The most common injury was the loss of fragment of the wing and tail membrane (< 1.0%), whereas the damage that occurred the least frequently was the hole in the wing membrane (< 0.5%).

**Fig 4:**
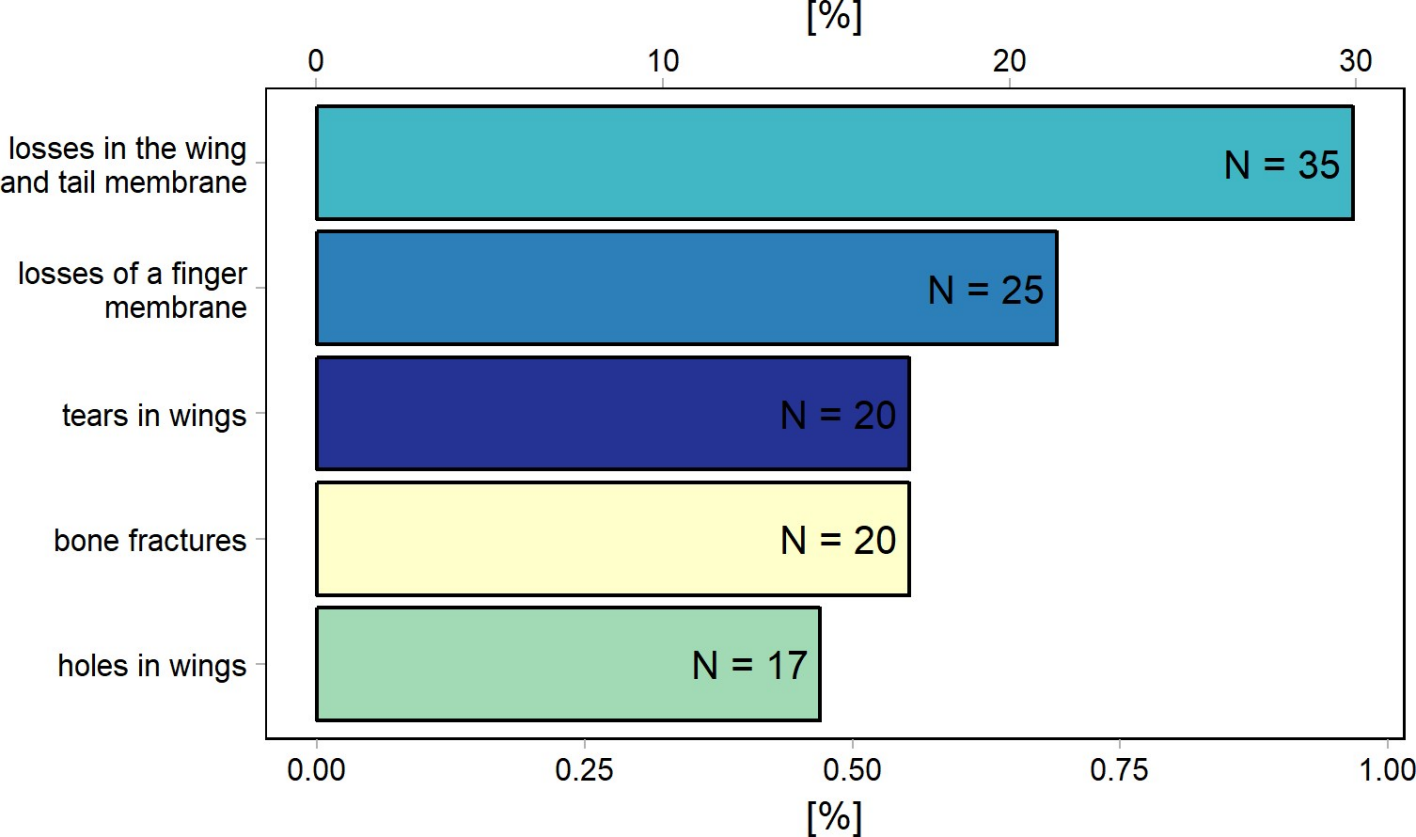
Proportion of damage types in all the bats studied. The upper axis shows the percentage of bats from each wing damage categories among all wing damages (N = 117). The lower axis shows the percentage of bats from each wing damage categories among all observed individuals (N = 3,525).

The analysis of the percentage of injuries to the membranes and the wings in the bats showed that the most frequently observed type of damage was loss of an edge of the wing membrane (29.3% of individuals with damages). We also found a high percentage of individuals with a fragment of a wing missing (21.6% of individuals with damages). Injuries classified as holes in wings were the least frequent (15% of individuals with damages) (Fig 4).

The frequency of occurrence and the locations of the damage to the wing membrane differed significantly among the studied bat species, which supports our first hypothesis. The largest number of injuries was observed in the case of Natterer’s bat (21.74% of individuals with damages), Daubenton’s bat (17% of individuals with damages), the lesser horseshoe bat (8.46% of individuals with damages), the greater mouse–eared bat (7.42% of individuals with damages), the western barbastelle (0.42% of individuals with damages) and the lowest for the brown long–eared bat (0.3% of individuals with damages). The frequency of each type of damage depends on species (Table 1).

**Table 1:** Results of the logistic regression for species, foraging mode, habitat type and foraging habitat and number of wings damages.

**Mod: damage ~ species, family = binomial**				
**Coefficients:**	Estimate	Std. Error	z value	Pr(>|z|)
**Intercept**	-3.47933	0.09908	-35.115	< 2e-16***
***B*. *barbastellus***	-1.98704	0.33202	-5.985	2.17e-09***
***M*. *daubentonii***	1.90583	0.21290	8.952	< 2e-16***
***M*. *myotis***	0.98016	0.21710	4.515	6.34e-06***
***M*. *nattereri***	2.19840	0.27149	8.098	5.61e-16***
***P*. *auritus***	-2.32580	1.00639	-2.311	0.020831*
***R*. *hipposideros***	1.09811	0.33035	3.324	0.000887***
**Mod: damage ~ (foraging mode) aerial hawking + gleaning foragers + passive gleaning foragers + trawling foragers, family = binomial**				
**Coefficients:**	Estimate	Std. Error	z value	Pr(>|z|)
**Intercept**	-3.48696	0.09346	-37.312	< 2e-16***
**Aerial hawking**	-1.32097	0.22928	-5.762	8.34e-09***
**Gleaning foragers**	0.69311	0.20468	3.386	0.000709***
**Passive gleaning foragers**	0.98779	0.21459	4.603	4.16e-06***
**Trawling foragers**	1.91345	0.21034	9.097	< 2e-16***
**Mod: damage ~ (habitat type) background cluttering + high cluttering + uncluttered, family = binomial**				
**Coefficients:**	Estimate	Std. Error	z value	Pr(>|z|)
**Intercept**	-3.41405	0.09436	-36.180	< 2e-16***
**Background-cluttering**	-0.40722	0.15263	-2.668	0.00763**
**High cluttering**	1.03282	0.32896	3.140	0.00169**
**Uncluttered**	1.84054	0.21075	8.733	< 2e-16***
**Mod: damage ~ (foraging habitats) edge space + narrow space, family = binomial**				
**Coefficients:**	Estimate	Std. Error	z value	Pr(>|z|)
**Intercept**	-3.477933	0.09908	-35.115	< 2e-16***
**Edge space**	-0.22582	0.16071	-1.405	0.15997
**Narrow space**	0.50331	0.18829	2.673	0.00752**

* p < 0.05

** p < 0.01

*** p < 0.001

In terms of the damage location, in the case of Natterer’s bat and the western barbastelle, the most common were injuries within the plagiopatagium. In the Daubenton’s bat, the greater mouse–eared bat, and the lesser horseshoe bat, the largest number of damages occurred within the dactylopatagium. Injuries to the uropatagium were observed the least often (1 greater mouse–eared bat and 1 lesser horseshoe bat). No injuries to the propatagium were found in any of the studied individuals, irrespective of the species (Table 2).

**Table 2:** Proportion of damage in various parts of the wings and the tail membrane in the studied bat species (I–tail membrane, uropatagium, II–wing membrane, plagiopatagium, III–finger membrane, dactylopatagium, IV–propatagium).

Parts of the wing	Mmyo		Mdau		Mnat		Bbar		Paur		Rhip	
	N	%	N	%	N	%	N	%	N	%	N	%
**I**	1	3.4										
**II**	11	37.9	9	26.5	13	65.0	5	50.0			4	36.4
**II/III**	2	6.9	5	14.7			1	10.0			1	9.1
**III**	15	51.7	20	58.8	7	35.0	4	40.0	1	100	5	45.5
**III/I**											1	9.1
**IV**												
**Total**	29	100	34	100	20	100	10	100	1	100	11	100

The abbreviation name: Rhip–*Rhinolophus hipposideros*, Mdau–*Myotis daubentonii*, Mnat–*Myotis nattereri*, Mmyo–*Myotis myotis*, Bbar–*Barbastella barbastellus*, Paur–*Plecotus auritus*.

The frequency of damage on wing membrane (II) and finger membrane (III) depends on foraging mode and habitat type of bat species (Table 3). These damages occurred most frequently in gleaning foragers, passive gleaning foragers and trawler foragers and in uncluttered habitat.

**Table 3:** Results of the logistic regression for foraging mode and location of the damage on wings.

**Mod: wing membrane, plagiopatagium (II) ~ (habitat type) background cluttering + high cluttering + uncluttered, family = binomial**				
**Coefficients:**	Estimate	Std. Error	z value	Pr(>|z|)
**Intercept**	-4.1592	0.1347	-30.884	< 2e-16***
**Background-cluttering**	-0.3243	0.2132	-1.521	0.128
**High cluttering**	0.9403	0.4755	1.977	0.048*
**Uncluttered**	1.5833	0.3082	5.137	2.79e-07***
**Mod: wing membrane, plagiopatagium (II) ~ (foraging mode) aerial hawking + gleaning foragers + passive gleaning foragers + trawling foragers, family = binomial**				
**Coefficients:**	Estimate	Std. Error	z value	Pr(>|z|)
**Intercept**	-4.2482	0.1346	-31.566	< 2e-16***
**Aerial hawking**	-1.3015	0.3307	-3.935	8.30e-05***
**Gleaning foragers**	0.8526	0.2748	3.102	0.00192**
**Passive gleaning foragers**	0.9024	0.3126	2.886	0.00390**
**Trawling foragers**	1.6724	0.3082	5.427	5.75e-08***
**Mod: wing membrane, plagiopatagium (II) ~ (foraging habitats) edge space + narrow space, family = binominal**				
**Coefficients:**	Estimate	Std. Error	z value	Pr(>|z|)
**Intercept**	-4.2172	0.1411	-29.897	< 2e-16***
**Edge space**	-0.1622	0.2249	-0.721	0.471
**Narrow space**	0.3898	0.2769	1.408	0.159
**Mod: wing membrane, plagiopatagium (III) ~ (habitat type) background cluttering + high cluttering + uncluttered, family = binomial**				
**Coefficients:**	Estimate	Std. Error	z value	Pr(>|z|)
**Intercept**	-3.9468	0.1215	-32.472	< 2e-16***
**Background-cluttering**	-0.5367	0.2052	-2.615	0.00891**
**High cluttering**	1.0805	0.4071	2.654	0.00796**
**Uncluttered**	2.0124	0.2461	8.178	2.89e-16***
**Mod: finger membrane, dactylopatagium (III) ~ (foraging mode) aerial hawking + gleaning foragers + passive gleaning foragers + trawling foragers, family = binomial**				
**Coefficients:**	Estimate	Std. Error	z value	Pr(>|z|)
**Intercept**	-4.0071	0.1198	-33.461	< 2e-16***
**Aerial hawking**	-1.3005	0.2935	-4.431	9.36e-06***
**Gleaning foragers**	0.4236	0.2879	1.471	0.141172
**Passive gleaning foragers**	0.9404	0.2755	3.413	0.000642***
**Trawling foragers**	2.0727	0.2452	8.453	< 2e-16***
**Mod: wing membrane, plagiopatagium (III) ~ (foraging habitats) edge space + narrow space, family = binomial**				
**Coefficients:**	Estimate	Std. Error	z value	Pr(>|z|)
**Intercept**	-4.0187	0.1218	-31.363	< 2e-16***
**Edge space**	-0.2447	0.2093	-1.169	0.2424
**Narrow space**	0.5283	0.2401	2.200	0.0278*

* p < 0.05

** p < 0.01

*** p < 0.001

Individuals with a single type of damage represented 89.52% of all the individuals with damages, two types of damages were found in 9.52% of the individuals. Bats with three different injuries were the least numerous (0.95%).

**Fig 5:**
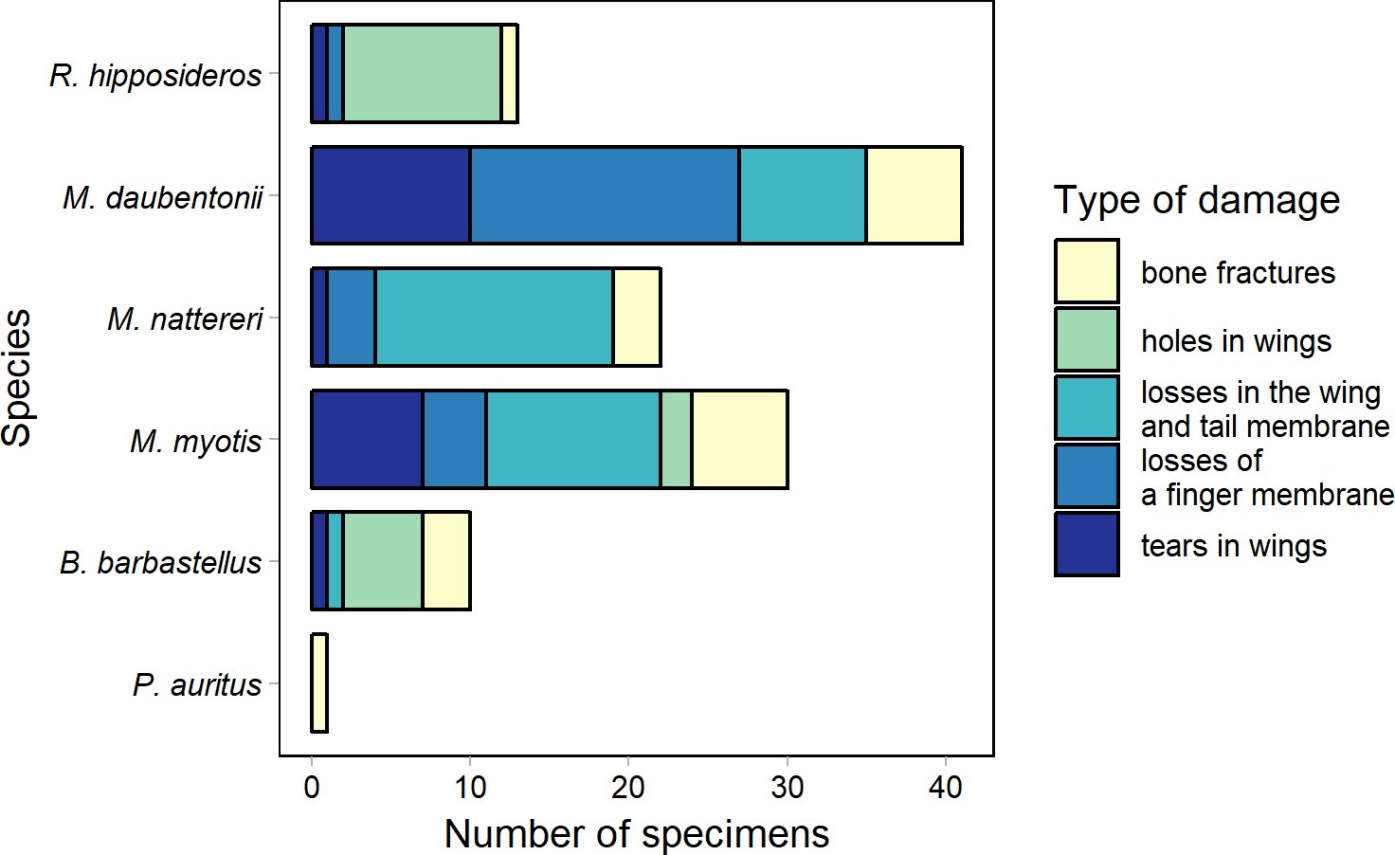
Frequencies of each type of membrane damage within a species. Number of specimens = 105, number of damages = 117.

We observed the differences between the injury groups in particular species. All types of damages to the wing membranes and the wings occurred only in greater mouse–eared bats. The largest number of injuries was the losses in the wing and tail membrane (11 of 29 individuals with injuries) (Fig 5). Losses in an edge of the wing membrane or scarred tears causing a deformation of the wings were most frequently found in Natterer’s bats (15 of 20 individuals with injuries). The most common wing injury in the Daubenton’s bat was the lack of a fragment of the wing (17 of 34 individuals with injuries). In the horseshoe and western barbastelle bats, wing holes were the most frequently recorded injuries (9 of 11 and 5 of 10 individuals with injuries, respectively).

Foraging mode of bat species determines number of wing and tail membrane damages. Most vulnerable to damages are trawling foragers. There is also a significant relation between number of damages and habitat type used by bat species, which occurred most frequently in bats flying in uncluttered habitat. Condition of wing and tail membrane is also related to type of foraging habitat. In this case, vulnerable to damages were bats foraging in narrow space (Table 1). These results support our second hypothesis, that ecological differences determine the number of damages of wings and tail membrane.

**Fig 6:**
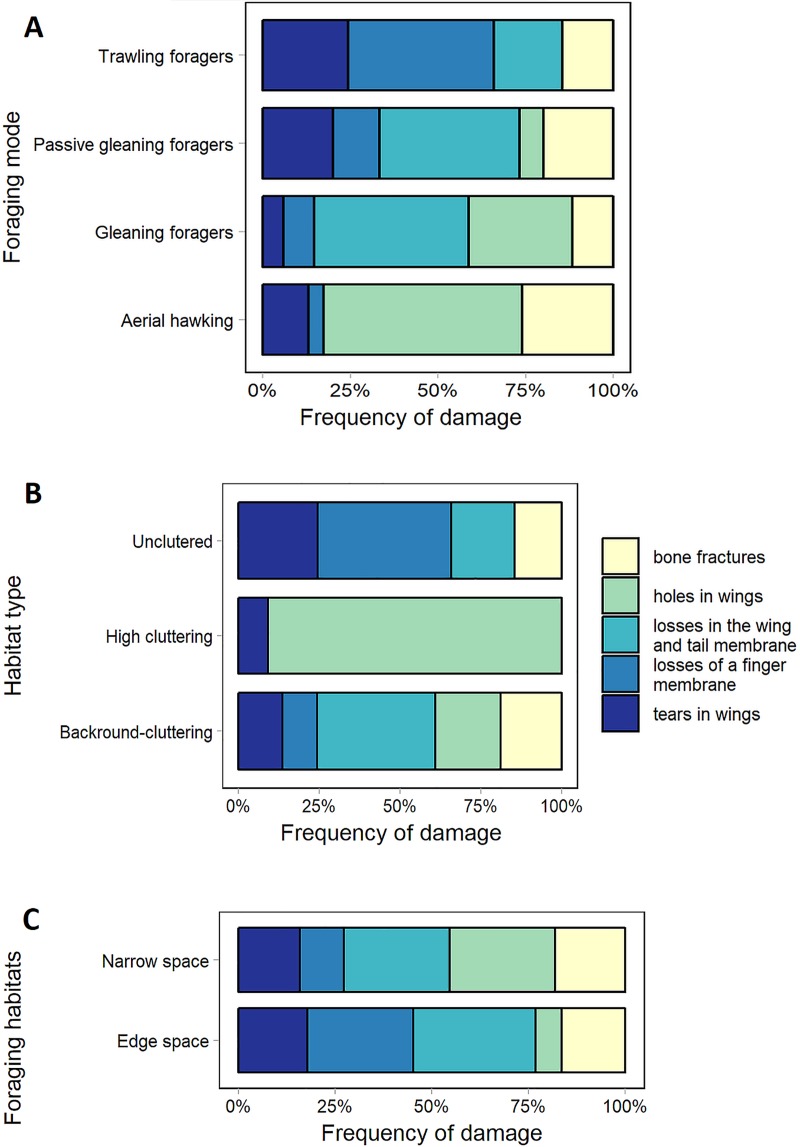
Occurrence of the damage in bat wings according on foraging mode (A), habitat type (B), foraging habitats (C).

Differences in occurrence of different type of damage between foraging mode guilds are statistically significant (Pearson’s χ^2^ test; χ^2^ = 62.443, df = 12, p-value = 8.065e-09), which supports our third hypothesis. In trawling foragers most common type is loss of finger membrane, in gleaning bats and passive gleaning foragers occurs losses of a wing and tail membrane. Aerial hawking bats most frequently have holes of wing (Fig 6A). Type of habitat used by bats also determine type of damage (Pearson’s χ^2^ test; χ^2^ = 61.618, df = 8, p-value = 2.242e-10). The biggest variety of damages occur in bats flying in uncluttered habitat (mostly losses of wing and tail membrane). Bats foraging in background-cluttering habitat have mostly bone fractures (Fig 6B). Choice of foraging habitat is another factor that influences differences in wing damages in bat species. Bats foraging in edge space area have more losses in the wing, tail and finger membranes. Species foraging in narrow space represent similar frequency of wing and tail membrane losses to previous guild and also high proportion of holes in the wings (Fig 6C).

## Discussion

This is the first study that describes the problem of damages in flight enabling body parts of European bat species. The research results indicated that the number of individuals with damaged wings was not large as compared to the total number of captured bats. In the presented research, bats with damage constituted less than 3% of the individuals. However, in some species, for example, Natterer’s bat, injuries to the wings may affect a significant number of individuals.

In bats of the Phyllostomidae family, only 0.6% individuals with damages were recorded [9]. In the case of pallid bats *Antrozous pallidus*, 41% of the population sustained damage [8]. The high percentage of bats with injures is noteworthy, especially that observations were made during one night only.

In the presented research, we found that the percentage of injuries in the form of holes in the wings was low (0.5%). This contradicted the opinion on the prevalence of such damage [8, 18]. In different phenological periods, the proportion of individuals with holes in the wings is greater. Further, these types of injuries heal quickly [11, 16, 17]. Only two cases of injuries within the uropatagium were found in the present study. Injuries in the tail membrane are very rare [14]. The uropatagium is the most durable part of a wing membrane [3]. This may be a reason that injuries in this part of the wing membrane occur infrequently, in general. At the same time, injuries in this part of the membrane heal even faster than those in the other parts [15, 26], which could explain the low probability of their identification.

The causes of wing injuries are difficult to determine. Some of the injuries may be caused during foraging. A feature that differentiates the bat species in question is the evolutionary adaptation to the field orientation and the method of echolocation, which also translates into the method of acquiring food [22] and thus, the probability of membrane injuries. Our study showed significant differences in occurrence and location of flight enabling body parts damages among species. This distinction is strongly related to their foraging behavior and ecology.

**Fig 7:**
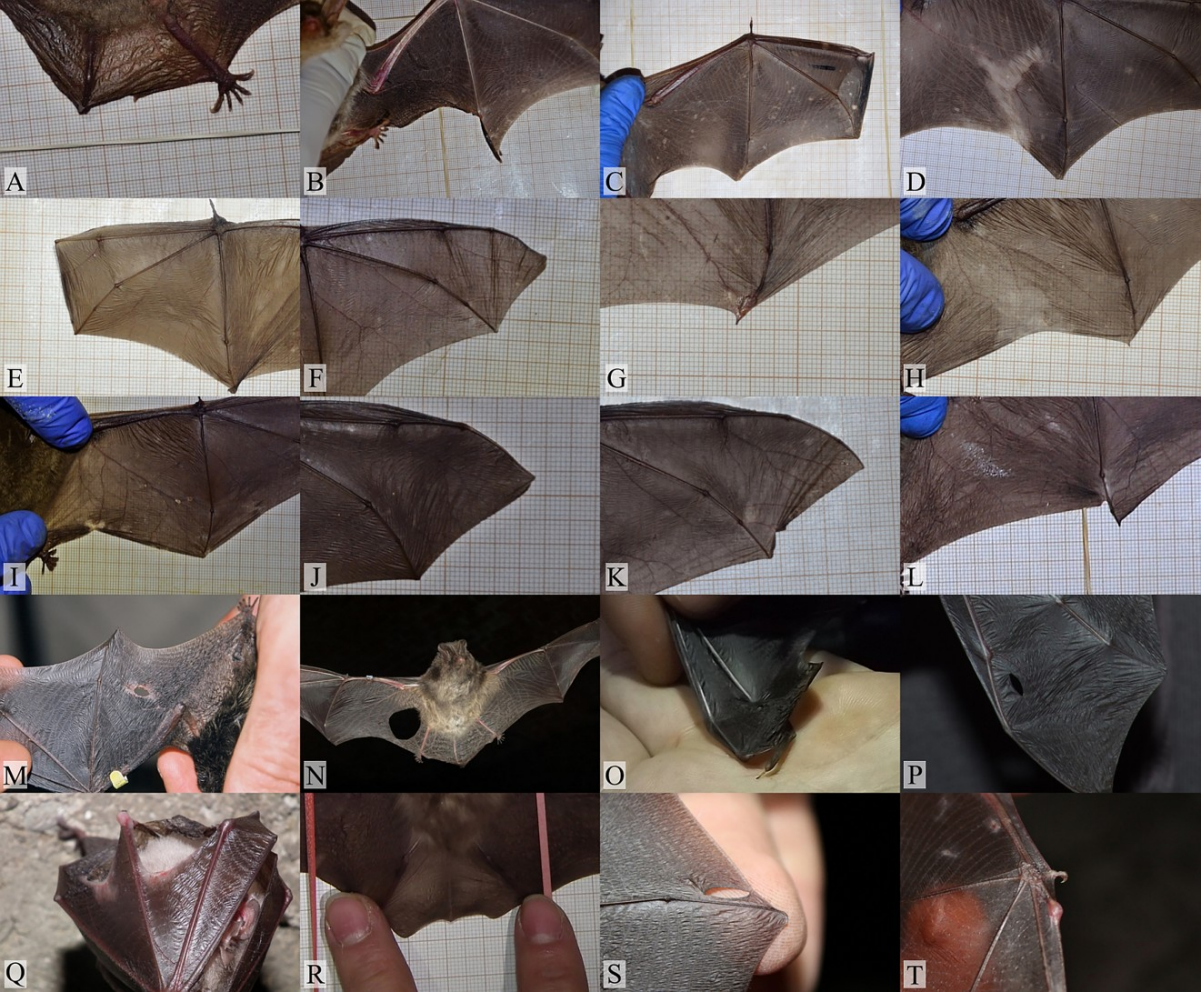
Damage to wings of bats. A–D greater mouse–eared bat, E–H Daubenton’s bat, I–L Natterer’s bat, M–P western barbastelle, Q–T lesser horseshoe bat. Tears in the wing–L, K. Holes in wings–M, N, P, S. Losses in the wing and tail membrane–A, B, D, I, R, Q, H. Loss of a finger membrane–C, E, F, J. Bone fractures—O, T, G.

The lowest number of injuries should occur in Daubenton’s bats, who most often hunt over open waters without vegetation, where there are few obstacles. Insects caught over water dominate in their diet [27, 28]. If necessary, Daubenton’s bats can change their feeding tactics, for example, they can collect their prey [29] or hunt in the forest and over wet meadows [30]. In the presented study, however, this species had the second highest number of injuries of the dactylopatagium. Some of the wing injuries were reminiscent of a “scissors cut” (Fig 7E). The most distal phalange segment is also the least mineralized part of the wing [3, 31–33]. The flexible tip of the wing may be more susceptible to injuries, for example, in the case of getting hit by or getting caught in fishing lines [34].

Natterer’s bat seems to be the most prone to collisions. It is regarded as a gleaning species. Its diet consists of a number of non–flying arthropods, such as spiders and harvestmen, the capture of whose may involve collisions with obstacles [35–38]. In our study, Natterer’s bat had the highest number of injuries to the plagiopatagium. All injuries in this species were small losses of the wing membrane (Fig 2D). Natterer’s bats have special type of hair at the end of the uropatagium, which may play a significant role during hunting [39]. In a laboratory study most of the capture attempts (62%) involved the tail membrane [36]. They also had similar findings during observations in the field. Natterer’s bat brushed the vegetation with its tail membrane [36]. In the presented research, we did not observe any damage to the uropatagium.

The diet of the lesser horseshoe bat and the brown long–eared bat consists mainly of dipterans and lepidoptera [40] and lepidoptera [38], respectively. The proportion of arthropods collected by gleaning is lower than that collected by other foraging techniques and only complements the diet. Another of the species studied, which collects its prey from the surface, is the greater mouse–eared bat. It had the fewest number of injuries among the three tested *Myotis* bat species. It chooses forests without undergrowth [41], where it hunts for ground beetles [42, 43]. It is a species that should have an increased number of injuries, just as Natterer’s bat. However, the foraging behavior of the greater mouse–eared bat is based on passive listening [22, 44]. During foraging they fly slowly above ground listening to sounds of their potential prey. This might be the reason for hitting less obstacles. The greater mouse–eared bat is also larger than other species researched in this study. Thus its wings and tail membrane are less dainty.

The lesser horseshoe bats differ in terms of the method of echolocation from the representatives of the Vespertilionidae family. Lesser horseshoe bats echolocate at very high frequencies [45] within a very small range [46]. Therefore, these bats avoid open spaces and move only among vegetation, trees, and other linear landscape elements [46, 47]. This species, like the barbastelle, feeds mainly in deciduous forests [46, 48, 49]. Despite some similarities in the diet and the selection of feeding grounds, the lesser horseshoe bat suffers injuries to wing membranes much more frequently than the barbastelle, as observed in the present study. However, it may fly more often in the treetops or among groups of trees [50] and is thus more likely to be exposed to potential injuries. During foraging, the western barbastelle bat flies near branches of trees, preferring man–made and natural forest clearings [51, 52].

In the case of the lesser horseshoe bat, holes in the wing membranes dominated over the other types of the injuries, in contrast to the other species studied. The predominance of this type of injury was also observed in the western barbastelle bat, but it was more than twice as rare as in the lesser horseshoe bat. This finding might be attributed to the different habitat in which both species hunt. Lesser horseshoe bats most often catch their prey with the wings but in more dense habitat than barbastelle bat [23]. They can collide with the obstacles as they hunt. This might explain why the most frequent injury found in this bat species was the damaged plagiopatagium and dactylopatagium.

In spite of the large number of western barbastelle bats examined, the recorded number of individuals with injuries was negligible. The diet of the western barbastelle bats consists mainly of moths and some other invertebrates, including spiders (Araneae) [38, 53], which is similar to the diet of the brown long–eared bat or the lesser horseshoe bat. However, the western barbastelle bat hunts mainly during flight, farther from vegetation than the two species mentioned earlier; therefore, it is less vulnerable to accidental collisions resulting in cuts in wings.

The lowest number of injuries occurred in the brown long–eared bat, which is characterized by a slow, fluttering but agile flight [54]. The existing literature reports only a few cases of damage to wings and wing membranes in this species [10, 14]. In order to collect its prey from the surface of various objects, the brown long–eared bat often passively listens, without using echolocation skills. Additionally, the brown long–eared bat can catch its prey in an open space. A majority of prey caught this way are moths. The bat catches them with its mouth and less often with the wing membranes [55]. In addition, the brown long–eared bat uses its eyes to a greater extent during foraging for food than the other bat species [30, 56], which, combined with the agile flight, may improve its ability to detect obstacles and thus avoid injuries.

The determination of the causes of damages to the wing membranes in bats is very difficult. Injuries may result from mechanical injuries, such as hooking on vegetation, for example, thorn shrubs [8], collisions with a barbed wire [57, 58], or getting caught up in fishing lines [34]. Damage to the wings can also be caused by sharp objects in the hideouts. These are all kinds of sharp elements such as nails, sharp ridges of metal sheets in attics and in hibernation places, garbage, or improperly performed entrance protections (according to the authors of this article, S2 Appendix). These elements may also pose a threat during the mating season. At the time of swarming, which is often accompanied by the so–called “flight displays”, individuals may be less attentive or more willing to take higher risks and thus be more likely to be exposed to wing membrane injuries [59–61]. Vulnerability for wing damages might be greater in dense habitat, with solid obstacles. The video of small *Myotis* species swarming in “Nietoperek” reserve (S2 Appendix) showed that bats were flying in damaged staircase, containing rambling metal bars. In the video it is possible to hear sounds of bats’ wings flapping on obstacles.

Damage to wing membranes may also be the effect of predation. In Central Europe, the predators hunting for bats are owls: tawny owl *Strix aluco* [62, 63], barn owl *Tyto alba* [64], and less frequently diurnal predatory birds Corvidae and Paridae [65–68]. Damage to the wing membranes and wings may also be caused by rodent predation [69], by another bat’s attack ([70, 71], own observations), by domestic cats [72, 73] or martes [74].

Despite their ability to hibernate, bats are exposed to the negative effects of low temperatures, i.e., frostbites. There were also cases of the affected fragments of wings in the common noctule bat *Nyctalus noctula* (I. Gottfried and T. Gottfried’s own observations).

In some cases, the injuries may be followed by secondary infections: bacterial, viral, or fungal [58]. Histopathological analyses suggest that *Pseudogymnoascus destructans* may cause wing damage [19, 20]. The healing period of the infected wing can then be slowed down [17]. As a result, the injury may have a negative effect on the functioning of the wing, particularly on the efficiency of foraging [13, 19].

Our results showed that the flight–enabling parts of a bat’s body are vulnerable to damage, and the bats themselves are liable to injuries. However, the scale of the injuries varies among species. Differences are related to foraging mode and ecology of species. More vulnerable are gleaning species such as *M*. *nattereri*. Other species such as *B*. *barbastellus* and *P*. *auritus* have few membrane injuries, which can be classified as accidental. A further survey should be conducted to explain whether wing injuries are caused by the hunting strategy and the echolocation type or are simply accidental events. We suggest that more gleaning bat species be investigated.

## Supporting information

S1 Appendix Number of individuals of bats without and with wing damage found in the years 2000–2016 in western and southern Poland. (DOCX)

S2 Appendix Bats swarming in damaged staircase, containing rambling metal bars, in “Nietoperek” reserve, western Poland. (MP4)

## References

[1] Swartz SM, Iriarte–Díaz J, Riskin DK, Breuer SK. A bird? A plane? No, it’s a bat: an introduction to the biomechanics of bat flight In: Gunnell GF, Simmons NB, editors. Evolutionary History of Bats: Fossils, Molecules and Morphology. Cambridge: University Press; 2012 pp. 317–352.

[2] Holbrook KA, Odland GF. A collagen and elastic network in the wing of the bat. J Anat. 1978;126: 21–36. . 649500 PMC1235709

[3] Swartz SM, Groves SM, Kim HD, Walsh WR. Mechanical properties of bat wing membrane skin. J Zool. 1996;239: 357–378. 10.1111/j.1469-7998.1996.tb05455.x

[4] Norberg UM, Rayner JMV. Ecological morphology and flight in bats (Mammalia; Chiroptera): wing adaptations, flight performance, foraging strategy and echolocation. Philos T Roy Soc Lond B. 1987;316(1179): 335–427. 10.1098/rstb.1987.0030

[5] Fenton MB, Bogdanowicz W. Relationships between external morphology and foraging behaviour: bats in the genus *Myotis*. Can J Zool. 2002;80: 1004–1013. 10.1139/z02-083

[6] Kalko EKV, Schnitzler HU. The echolocation and hunting behavior of Daubenton’s bat, *Myotis daubentoni*. Behav Ecol Sociobiol. 1989;24(4): 225–238. 10.1007/BF00295202

[7] Schmieder DA, Zsebők S, Siemers BM. The tail plays a major role in the differing maneuverability of two sibling species of mouse–eared bats (*Myotis myotis* and *Myotis blythii*). Can J Zool. 2014;92: 965–977. 10.1139/cjz-2014-0104

[8] Davis R. Wing defects in a population of pallid bats. Am Midl Nat. 1968;79: 388–395. 10.2307/2423184

[9] Castillo–Figueroa D, Pérez–Torres J. First records of wing defects in phyllostomid bats from Colombia. J Bat Res Conserv. 2018;11(1). 10.14709/BarbJ.11.1.2018.01

[10] Stebbings RE. A population study of bats of the Genus *Plecotus*. J Zool. 1966;150: 53–75. 10.1111/j.1469-7998.1966.tb02998.x

[11] Davis R, Doster SE. Wing repair in pallid bats. J Mammal. 1972;53: 377–378. 10.2307/1379180

[12] Bogdanowicz W, Urbańczyk Z. A case of self–broken forearm in *Myotis brandtii* (Eversmann, 1845). Acta Theriol. 1986;31: 180–181. 10.4098/AT.arch.86-19

[13] Voigt ChC. Bat flight with bad wings: is flight metabolism affected by damaged wings? J Exp Biol. 2013;216: 1516–1521. 10.1242/jeb.079509 23348945

[14] Mulder J, Janssen R. Tail defects in two Daubenton’s bats (*Myotis daubentonii*). Lutra 2016;59: 111–114.

[15] Faure PA, Re DE, Clare EL. Wound healing in the flight membranes of big brown bats. J Mammal. 2009;90: 1148–1156. 10.1644/08-MAMM-A-332.1

[16] Weaver KN, Alfano SE, Kronquist AR, Reeder DM. Healing rates of wing punch wounds in free–ranging little brown myotis (*Myotis lucifugus*). Acta Chiropterol. 2009;11: 220–223. 10.3161/150811009X465866

[17] Ceballos–Vasquez A, Caldwell JR, Faure PA. Seasonal and reproductive effects on wound healing in the flight membranes of captive big brown bats. Biol Open. 2015;4: 95–103. 10.1242/bio.201410264 25527646 PMC4295170

[18] Wing punch and hair sampling protocols, American Museum of Natural History. 2018. http://research.amnh.org/vz/mammalogy/donating-bat-tissue-and-hair-samples-genomic-and-stable-isotope-studies/wing-punch-and-hair-sampling

[19] Reichard JD, Kunz TH. White–nose syndrome inflicts lasting injuries to the wings of little brown myotis (*Myotis lucifugus*). Acta Chiropterol. 2009;11: 457–464. 10.3161/150811009X485684

[20] Fuller NW, Reichard DJ, Nabhan ML, Fellows SR, Pepin LC, Kunz TH. Free–ranging little brown myotis (*Myotis lucifugus*) heal from wing damage associated with white–nose syndrome. EcoHealth. 2011;8(2): 154–162. 10.1007/s10393-011-0705-y 21922344

[21] Cichocki J, Stopczyński M, Bator A, Grzywiński W, Ignaczak M, Ignaszak K, et al. The number of bats wintering in the Nietoperek bat reserve in 2015. In: XXIV National Bat Conference. Kazimierz Dolny. Poland. In: XXIV, National Bat Conference. Kazimierz Dolny. Poland. Book of Abstracts. 2015; pp. 36. (in Polish full English summary). https://www.researchgate.net/publication/285236621.

[22] Denzinger A, Schnitzler H–U. Bat guilds, a concept to classify the highly diverse foraging and echolocation behaviors of microchiropteran bats. Front Physiol. 2013;4: 164 10.3389/fphys.2013.00164 23840190 PMC3699716

[23] Dietz C, Kiefer A. Bats of Britain and Europe. London: Bloomsbury; 2016.

[24] Grace JB, Johnson DJ, Lefcheck JS, Byrnes JEK. Quantifying relative importance: computing standardized effects in models with binary outcomes. Ecosphere. 2018;9(6):e02283 10.1002/ecs2.2283

[25] R Core Team. R: a language and environment for statistical computing. Vienna: R Foundation for Statistical Computing; 2018.

[26] Pollock T, Moreno ChR, Sánchez L, Ceballos–Vasquez A, Faure PA, Mora EC. Wound healing in the flight membranes of wild big brown bats. J Wildlife Manage. 2016;80: 19–26. 10.1644/08-MAMM-A-332.1

[27] Flavin DA, Biggane SS, Shiel CB, Smiddy P, Fairley JS. Analysis of the diet of Daubenton’s bat *Myotis daubentonii* in Ireland. Acta Theriol. 2001;46: 43–52. 10.1007/BF03192415

[28] Vesterinen EJ, Lilley T, Laine VN, Wahlberg N. Next generation sequencing of fecal DNA reveals the dietary diversity of the widespread insectivorous predator Daubenton’s bat (*Myotis daubentonii*) in southwestern Finland. Plos One. 2013;8(11): e82168 10.1371/journal.pone.0082168 24312405 PMC3842304

[29] Nissen H, Krüger F, Fichtner A, Sommer RS. Local variability in the diet of Daubenton’s bat (*Myotis daubentonii*) in a lake landscape of northern Germany. Folia Zool. 2013;62(1): 36–41. 10.25225/fozo.v62.i1.a5.2013

[30] Dietz C, Nill D, von Helversen O. Bats of Britain, Europe and Northwest Africa. London: A & C Black; 2009.

[31] Norberg UM. Bat wing structures important for aerodynamics and rigidity (Mammalia, Chiroptera). Z Morphol Tiere. 1972;73(1): 45–61. 10.1007/BF00418147

[32] Papadimitriou HM, Swartz SM, Kunz TH. Ontogenetic and anatomic in mineralization of the wing skeleton of the Mexican free–tailed bat, *Tadarida brasiliensis*. J Zool. 1996;240: 411–426. 10.1111/j.1469-7998.1996.tb05295.x

[33] Swartz SM, Middleton KM. Biomechanics of the bat limb skeleton: scaling, material properties and mechanics. Cells Tissues Organs. 2008;187: 59–84. 10.1159/000109964 18160803

[34] Sleeman DP. Fly fishing accidents and bats. Irish Nat J. 2013;32(2): 138–141. JSTOR, https://www.jstor.org/stable/24394432

[35] Arlettaz R. Foraging behaviour of the gleaning bat *Myotis nattereri* (Chiroptera, Vespertilionidae) in the Swiss Alps. Mammalia. 1996;60: 181–186. 10.1515/mamm.1996.60.2.181

[36] Swift SM, Racey PA. Gleaning as foraging strategy in Natterer’s bat *Myotis nattereri*. Behav Ecol Sociobiol. 2002;52: 408–416. 10.1007/s00265-002-0531-x

[37] Siemers BM, Swift MS. Differences in sensory ecology contribute to resource partitioning in the bats *Myotis bechsteinii* and *Myotis nattereri* (Chiroptera: Vespertilionidae). Behav Ecol Sociobiol. 2006;59: 373–380. 10.1007/s00265-005-0060-5

[38] Andreas M, Reiter A, Benda P. Dietary composition, resource partitioning and trophic niche overlap in three forest foliage–gleaning bats in Central Europe. Acta Chiropterol. 2012;14: 335–345. 10.3161/150811012X661657

[39] Czech NU, Klauer G, Dehnhardt G, Siemers BM. Fringe for foraging? Histology of the bristle–like hairs on the tail membrane of the gleaning bat, *Myotis nattereri*. Acta Chiropterol. 2008;10(2): 303–311. 10.3161/150811008X414872

[40] Ahmim M, Moali A. The diet of four species of horseshoe bat (Chiroptera: Rhinolophidae) in a mountainous region of Algeria: evidence for gleaning. Hystrix. 2013;24(2): 174–176. 10.4404/hystrix-24.2-8728

[41] Zahn A, Haselbach H, Güttinger R. Foraging activity of central European *Myotis myotis* in a landscape dominated by spruce monocultures. Mammal Biol. 2004;70(5): 265–270. 10.1016/j.mambio.2004.11.020

[42] Pereira MJR, Rebelo H, Rainho A, Palmeirim JM. Prey selection by *Myotis myotis* (Vespertilionidae) in a Mediterranean Region. Acta Chiropterol. 2002;4: 183–193. 10.3161/001.004.0207

[43] Zahn A, Rottenwallner A, Güttinger R. Population density of the greater mouse–eared bat (*Myotis myotis*), local diet composition and availability of foraging habitats. J Zool. 2006;269: 486–493. 10.1111/j.1469-7998.2006.00081.x

[44] Russo D, Jones G, Arlletaz R. Echolocation and passive listening by foraging mouse–eared bats *Myotis myotis* and *M*. *blythii*. J Exp Biol. 2007;210: 166–176. 10.1242/jeb.02644 17170159

[45] Jones G, Rayner J. Foraging behavior and echolocation of wild horseshoe bats *Rhinolophus ferrumequinum* and *R*. *hipposideros* (Chiroptera, Rhinolophidae). Behav Ecol Sociobiol. 1989;25: 183–191. 10.1007/BF00302917

[46] Motte G, Libois R. Conservation of the lesser horseshoe bat (*Rhinolophus hipposideros* Bechstein, 1800) (Mammalia: Chiroptera) in Belgium. A case study of feeding habitat requirements. Belg J Zool. 2002;132(1): 47–52. https://www.researchgate.net/publication/237772500

[47] McAney CM, Fairley JS. Habitat preference and overnight and seasonal variation in the foraging activity of lesser horseshoe bats. Acta Theriol. 1988;33(28): 393–402. https://rcin.org.pl/Content/11297

[48] Holzhaider J, Kriner E, Bernd–Ulrich R., Zahn A. Radio–tracking a lesser horseshoe bat (*Rhinolophus hipposideros*) in Bavaria: an experiment to locate roosts and foraging sites. Myotis. 2002;40: 47–54. www.swild.ch/rhinolophus/Holzhaider_Myotis2002.pdf

[49] Boughey K, Lake I, Haysom K, Dolman P. Effects of landscape–scale broadleaved woodland configuration and extent on roost location for six bat species across the UK. Biol Conserv. 2011;144: 2300–2310. 10.1016/j.biocon.2011.06.008

[50] Arlettaz R, Godat S, Meyer H. Competition for food by expanding pipistrelle bat populations (*Pipistrellus pipistrellus*) might contribute to the decline of lesser horseshoe bats (*Rhinolophus hipposideros*). Biol Conserv. 2000;93(1): 55–60. 10.1016/S0006-3207(99)00112-3

[51] Russo D, Luca C, Gareth J, Mazzoleni S. Roost selection by barbastelle bats (*Barbastella barbastellus*, Chiroptera: Vespertilionidae) in beech woodlands of central Italy: consequences for conservation. Biol Conserv. 2004;117: 73–81. 10.1016/S0006-3207(03)00266-0

[52] Hillen J, Kaster T, Pahle J, Kiefer A, Elle O, Griebeler ME, et. al Sex–specific habitat selection in an edge habitat specialist, the western barbastelle bat. Ann Zool Fen. 2011;48: 180–190. 10.5735/086.048.0306

[53] Rydell J, Natuschke G, Theiler A, Zingg PE. Food habits of the barbastelle bat *Barbastella barbastellus*. Ecography. 1996;19(1): 62–66. 10.1111/j.1600-0587.1996.tb00155.x

[54] Norberg UM. Aerodynamics, kinematics, and energetics of horizontal flapping flight in the long–eared bat *Plecotus auritus*. J Exp Biol. 1976;65: 179–212. https://jeb.biologists.org/content/65/1/179 993701 10.1242/jeb.65.1.179

[55] Anderson ME, Racey PA. Feeding behaviour of captive brown long–eared bats, *Plecotus auritus*. Anim Behav. 1991;42: 489–493. 10.1016/S0003-3472(05)80048-X

[56] Eklöf J, Jones G. Use of vision in prey detection by brown long–eared bats, *Plecotus auritus*. Anim Behav. 2003;66: 949–953. 10.1006/anbe.2003.2272

[57] Hinkel A, Rackow W. Unfalle von Fledermausen auf Kletten, Kakteen oder Stacheldraht. Nyctalus. 1994;5: 3–10.

[58] Mühldorfer K, Speck S, Kurth A, Lesnik R, Freuling C, Müller T, et al Diseases and causes of death in European bats: dynamics in disease susceptibility and infection rates. Plos One. 2011;6(12): e29773 10.1371/journal.pone.0029773 22216354 PMC3247292

[59] Parsons KN, Jones G, Greenaway F. Swarming activity of temperate zone microchiropteran bats: effects of season, time of night and weather conditions. J Zool. 2003;261: 253–254. 10.1017/S0952836903004199

[60] Veith M, Beer N, Kiefer A, Johannesen J, Seitz A. The role of swarming sites for maintaining gene flow in brown long–eared bat (*Plecotus auritus*). Heredity. 2004;93: 342–349. 10.1038/sj.hdy.6800509 15241447

[61] Gottfried I. Use of underground hibernacula by the barbastelle (*Barbastella barbastellus*) outside the hibernation season. Acta Chiropterol. 2009;11(2): 363–373. 10.3161/150811009X485594

[62] Obuch J. The representation of bats (Chiroptera) in the diet of owls (Strigiformes) (in Slovak). Vespertilio. 1998;3: 65–74. https://netopiere.sk/media/upload/3_10.pdf

[63] Lesiński G, Gryz J, Kowalski M. Bat predation by tawny owls *Strix aluco* in differently human‐transformed habitats. It J Zool. 2009;76: 415–421. 10.1080/11250000802589535

[64] Sommer RS, Niederle M, Labes R, Zoller H. Bat predation by the barn owl Tyto alba in a hibernation site of bats. Folia Zool. 2009;58(1): 98–103. https://www.ivb.cz/folia_zoologica/archive/58_98-103.pdf

[65] Radzicki G, Hejduk J, Bańbura J. Tits (*Parus major* and *Parus careleus*) preying upon hibernating bats. Ornis Fen. 1999;76:93–94. https://lintulehti.birdlife.fi/#/pdfhakucrit

[66] Obuch J. Food of the raven (*Corvus corax*) in Slovakia. Tichodroma. 2007;19: 1–10. https://www.tichodroma.sk/Tichodroma_19/Tichodroma_19.1-10.p.

[67] Estók P, Zsebők S, Siemers BM. Great tits search for, capture, kill and eat hibernating bats. Biol Lett. 2010;6(1):59–62. 10.1098/rsbl.2009.0611 19740892 PMC2817260

[68] Mikula P, Morelli F, Lučan RK, Jones DN, Tryjanowski P. Bats as prey of diurnal birds: a global perspective. Mammal Rev. 2015;46: 160–174. 10.1111/mam.12060

[69] Haarsma AJR, Kaal R. Predation of wood mice (*Apodemus sylvaticus*) on hibernating bats. Popul Ecol. 2016;58: 567–576. 10.1007/s10144-016-0557-y

[70] Piksa K. Aggressive behaviour of greater mouse–eared bat (*Myotis myotis*) towards lesser horseshoe bats (*Rhinolophus hipposideros*) in a hibernaculum. Acta Chiropterol. 2006;8: 566–571. 10.3161/1733-5329(2006)8[566:ABOGMB]2.0.CO;2

[71] Łupicki D, Cichocki J, Szkudlarek R, Ważna A. Cannibalism in maternity colonies of the greater mouse–eared bat *Myotis myotis*. Mammalia. 2010;74: 339–341. 10.1515/mamm.2010.031

[72] Ancillotto L, Serangeli TM, Russo D. Curiosity killed the bat: Domestic cats as bat predators. Mammal Biol. 2013;78(5): 369–373. 10.1016/j.mambio.2013.01.003

[73] Khayat ROS, Shaw KJ, Dougill G, Melling LM, Ferris GR, Cooper G, Grant RA. Characterizing wing tears in common pipistrelles (*Pipistrellus pipistrellus*): investigating tear distribution, wing strength, and possible causes. J Mammal. 2019;20(10):1–13. 10.1093/jmammal/gyz081 PMC6660809 31379390

[74] Urbańczyk Z. Fledermäuse (Chiroptera) in der Nahrung der Marders (*Martes* sp.). Säugetierkund Mitt. 1981;29: 77–79.

