# Supplementary material for: Frequent or scarce? Damage to flight–enabling body parts in bats (Chiroptera)
Source: PLoS One. 2019 Jul 22;14(7):e0219783. doi: 10.1371/journal.pone.0219783 (PMC6645484; doi:10.1371/journal.pone.0219783)
Supplement: S1 Appendix — (DOCX) [file pone.0219783.s001.docx]

**Appendix I. Number of individuals of bats without and with wing damage found in the years 2000-2016 in western and southern Poland.**

| **Localities** | **Year** | **Bat species** | **Individuals without damages** | **Individuals with damages** |
| --- | --- | --- | --- | --- |
| **Nietoperek reserve** | 2015 | *Myotis daubentonii* | 110 | 16 |
|  | 2015 | *Myotis nattereri* | 39 | 11 |
|  | 2015 | *Myotis myotis* | 115 | 13 |
|  | 2016 | *Myotis daubentonii* | 56 | 18 |
|  | 2016 | *Myotis nattereri* | 33 | 9 |
|  | 2016 | *Myotis myotis* | 75 | 13 |
| **Chłodnia (ice house) in Cieszków** | 2000 | *Myotis myotis* | 10 | 0 |
|  | 2001 | *Myotis myotis* | 12 | 0 |
|  | 2002 | *Myotis myotis* | 12 | 0 |
|  | 2003 | *Myotis myotis* | 15 | 0 |
|  | 2004 | *Myotis myotis* | 23 | 0 |
|  | 2005 | *Myotis myotis* | 27 | 0 |
|  | 2006 | *Myotis myotis* | 18 | 0 |
|  | 2008 | *Myotis myotis* | 14 | 1 |
|  | 2012 | *Myotis myotis* | 33 | 2 |
|  | 2015 | *Myotis myotis* | 11 | 0 |
|  | 2000 | *Plecotus auritus* | 24 | 0 |
|  | 2001 | *Plecotus auritus* | 31 | 0 |
|  | 2002 | *Plecotus auritus* | 24 | 0 |
|  | 2003 | *Plecotus auritus* | 61 | 0 |
|  | 2004 | *Plecotus auritus* | 43 | 1 |
|  | 2005 | *Plecotus auritus* | 32 | 0 |
|  | 2006 | *Plecotus auritus* | 25 | 0 |
|  | 2008 | *Plecotus auritus* | 15 | 0 |
|  | 2012 | *Plecotus auritus* | 65 | 0 |
|  | 2015 | *Plecotus auritus* | 12 | 0 |
|  | 2000 | *Barbastella barbastellus* | 89 | 0 |
|  | 2001 | *Barbastella barbastellus* | 166 | 0 |
|  | 2002 | *Barbastella barbastellus* | 219 | 3 |
|  | 2003 | *Barbastella barbastellus* | 494 | 1 |
|  | 2004 | *Barbastella barbastellus* | 247 | 0 |
|  | 2005 | *Barbastella barbastellus* | 255 | 0 |
|  | 2006 | *Barbastella barbastellus* | 531 | 1 |
|  | 2008 | *Barbastella barbastellus* | 178 | 1 |
|  | 2012 | *Barbastella barbastellus* | 149 | 1 |
|  | 2015 | *Barbastella barbastellus* | 38 | 3 |
| **Wiślańska Cave** | 2014 | *Rhinolophus hipposideros* | 55 | 7 |
| **Grabowa Cave** | 2014 | *Rhinolophus hipposideros* | 0 | 1 |
| **Cave in Stołów** | 2014 | *Rhinolophus hipposideros* | 3 | 1 |
| **Grodziec** | 2014 | *Rhinolophus hipposideros* | 30 | 1 |
|  | 2016 | *Rhinolophus hipposideros* | 31 | 1 |
| **Total** | | | **3420** | **105** |
